# Postoperative Morbidity and Factors Predicting the Development of Lymphoceles Following Lateral Pelvic Node Dissection for Rectal Cancer: A Cohort Study

**DOI:** 10.1245/s10434-024-16320-7

**Published:** 2024-10-24

**Authors:** Joseph Mathew, Mufaddal Kazi, Ashwin Desouza, Avanish Saklani

**Affiliations:** 1 Department of GI Surgical Oncology and Minimal Access Surgery, HealthCare Global Enterprises Ltd (HCG), Bangalore, India; 2 Division of Colorectal Oncology, Department of Surgical Oncology, Tata Memorial Centre, Mumbai, India

**Keywords:** Rectal cancer, Lateral pelvic lymph nodes, Total mesorectal excision, Lateral pelvic lymph node dissection, Morbidity, Pelvic lymphocele, Risk factors

## Abstract

**Purpose:**

Lateral pelvic node dissection (LPLND) is indicated in the surgical management of clinically significant pelvic lymphadenopathy associated with rectal malignancies. However, procedure-related morbidity, including the incidence and predisposing factors for lymphoceles arising in this setting have not been adequately evaluated.

**Methods:**

This retrospective single-institution study included 183 patients with nonmetastatic, lateral node-positive rectal cancer undergoing total mesorectal excision with LPLND between June 2014 and May 2023 to determine the incidence and severity of postoperative complications using the Clavien-Dindo system, with logistic regression performed to model a relationship between lymphocele-development and potentially-predictive variables.

**Results:**

In this cohort, mean age was 45.3 ± 12.81 years, 62.8% were male, and 27.9% had body mass index ≥ 25 kg/m^2^. Median tumor-distance from the verge was 3.0 (interquartile range [IQR] 1.0–5.0) cm. Following radiotherapy in 86.9%, all patients underwent surgery: 30.1% had open resection and 26.2% had bilateral LPLND. Median nodal-yield was 6 (IQR 4–8) per side. Postoperatively, 45.3% developed complications, with 18% considered clinically significant. Lymphoceles, detected in 21.3%, comprised the single-most common sequelae following LPLND, 46.2% arising within 30 days of surgery and 33.3% requiring intervention. On multivariate analyses, obesity (hazard ratio [HR] 2.496; 95% confidence interval [CI] 1.094–5.695), receipt of preoperative radiation (HR 10.026; 95% CI 1.225–82.027), open surgical approach (HR 2.779; 95% CI 1.202–6.425), and number of harvested nodes (HR 1.105; 95% CI 1.026–1.190) were significantly associated with lymphocele-development.

**Conclusions:**

Pelvic lymphoceles and its attendant complications represent the most commonly encountered morbidity following LPLND for rectal cancer, with obesity, neoadjuvant radiotherapy, open surgery, and higher nodal-yield predisposing to their development.

Lateral pelvic lymph node dissection (LPLND) is essential for the surgical staging and prognostication of genitourinary malignancies and has been considered therapeutic in the presence of isolated lateral pelvic lymphadenopathy regarded as locoregional disease in this setting.^1,2^ On the contrary, clinically significant lateral pelvic nodes (LPLNs), occurring in up to 25% of patients with primary adenocarcinoma of the rectum, was historically considered to represent systemic disease.^3,4^ However, recent studies have reported improvements in local control and stage-specific recurrence-free survival with LPLND in these patients.^5^ As a result, the procedure has gained acceptance in the surgical management of locally advanced rectal cancer (LARC), either upfront or “selectively” following chemoradiotherapy.^6,7^ In approximately 3–7% of cases, squamous cell carcinomas, neuroendocrine tumors, and melanomas may also arise from the anorectum and present with clinically significant LPLNs necessitating LPLND either per primum or when persisting after neoadjuvant therapy.^8^

Regardless of tumor histology or the primary organ system involved, the complex neurovascular anatomy of the pelvic sidewall makes LPLND a technically challenging procedure associated with greater intraoperative blood loss, longer operating duration, and higher complication rates, including significant functional deficits and genitourinary adverse effects impairing quality of life.^9,10^ Additionally, in contrast with gynecological and urological malignancies, the receipt of chemoradiotherapy in patients with LPLN-positive rectal primaries combined with a more extensive lymphadenectomy template, involving dissection beyond the obturator nerve up to the sacral nerve roots, adds to procedure-associated morbidity. Among the complications encountered, lymphoceles represent the single most common post-LPLND sequelae and, as an entity, is unique to pelvic lymphadenectomy, resulting from undrained fluid collections enclosed within the medially dissected ureterohypogastric fascia and the pelvic sidewall, with a propensity to develop complications in up to 10% of patients on account of secondary infection or mass effects on adjacent anatomical structures, manifesting as pelvic pain secondary to nerve impingement, obstructive uropathy, lower limb lymphedema, venous thrombosis, or intestinal obstruction.^11–17^ However, few studies have attempted to characterize the morbidity profile of LPLND postneoadjuvant radiation for rectal cancer or determine the factors predisposing to the formation of lymphoceles in this setting.^18,19^

This study was conducted to evaluate the incidence and severity of postoperative complications encountered in the early, intermediate, and late postoperative periods following LPLND and total mesorectal excision (TME) for rectal cancers associated with significant LPLNs, and to further identify the factors predisposing to the development of lymphoceles.

## Methods

This cohort study was conducted in the Colorectal Division of the Department of Surgical Oncology at a tertiary cancer center between June 2014 and May 2023 using data from the prospectively maintained Institutional database. Consecutive patients with nonmetastatic histopathologically proven rectal cancer with significant lateral pelvic lymphadenopathy, having undergone curative-intent resection of the primary with TME and LPLND at the index surgery, regardless of the receipt of chemoradiotherapy were selected. Patients with a history of having undergone pelvic surgery or radiation in the past were excluded, as were those requiring resections extending beyond a conventional TME (which included extended-TME, wherein partial resections of involved anatomical structures and/or organs were performed, and beyond-TME, involving adjacent organ resections and exenterations), since these procedures had the potential to adversely affect postoperative recovery and genitourinary function, resulting in a varied morbidity profile.

Following standard evaluation for rectal cancer, including history and physical examination, tumor markers, colonoscopy and biopsy, MRI pelvis, and contrast-enhanced CT scans of the thorax and abdomen, all patients were treated as per the decision of the multidisciplinary tumor board. On imaging, as reviewed by two radiologists, clinically significant lateral pelvic nodal metastases were defined as obturator or (internal, external, or common) iliac lymphadenopathy with short-axis diameter ≥ 7 mm on the index scan or ≥ 4 mm (for internal iliac nodes) and ≥ 6 mm (for the other nodal groups) following neoadjuvant therapy, and/or with suspicious morphology, including rounded-shape, ill-defined borders or heterogenous signal intensities.^20^

In patients with adenocarcinoma histology, the presence of significant LPLNs was regarded as LARC and an indication for neoadjuvant therapy delivered either as long-course chemoradiotherapy (LCCRT) or short-course RT (SCRT) with consolidation chemotherapy^21,22^ as per the decision of the joint clinic. The lateral pelvic nodal basins were also included in the radiation portal with boost given at the discretion of the treating physician. Patients with other histologies also underwent multidisciplinary evaluation, anorectal melanomas undergoing upfront resection, and neuroendocrine carcinomas considered for platinum-based neoadjuvant chemotherapy with or without radiation, whereas surgery in squamous cell carcinoma was considered a salvage procedure for residual or recurrent disease following definitive chemoradiotherapy.

Curative-intent surgery was performed 8–12 weeks postradiotherapy, with the type of surgery and approach decided by a panel of surgeons based on extent of disease and radiological response to therapy. All patients underwent open or minimally invasive (laparoscopic or robotic) TME with LPLND on the side(s) affected, the procedure and template having been standardized and described previously, and the boundaries of dissection remaining unchanged regardless of histology.^23^ No attempt was made to obliterate the resultant LPLND cavity with autologous flaps or tissue sealants in any of the patients. The colorectal surgeons involved in the study were experienced in all three surgical approaches and were beyond the learning curve for LPLND.^24^

Patients were followed-up 3-monthly during the first 2 years after treatment completion and 6-monthly thereafter. Contrast-enhanced CT of the abdomen and pelvis was performed 6 months following surgery involving LPLND, apart from the standard annual CECT of the thorax, abdomen, and pelvis recommended in the surveillance of resected colorectal cancer. The onset of adverse symptoms or evidence of abnormal clinical and/or biochemical findings on follow-up was considered an indication for earlier imaging, the modality dependent on the patient’s history and clinical presentation.

Lymphoceles were diagnosed on CECT as well-defined, water-density, uniloculated, or septate fluid collections developing along the pelvic sidewall in the remnant post-LPLND cavity or on ultrasound as anechoic fluid collections alongside the vessels in the para-iliac region of the lateral pelvic compartment with or without septations or internal echoes.^25^ Any secondary complications, including superadded infection, hemorrhage into a collection, or adjacent organ impingement resulting in chronic pelvic pain, urinary tract obstruction, or lymphovascular insufficiency (deep vein thrombosis or lymphedema), were recorded and correlated clinically. Collections arising in the central pelvis were excluded.

The preoperative parameters analyzed included clinicodemographic data, performance status, body mass index (BMI), neoadjuvant therapy received, and schedule. Obesity was defined as BMI ≥ 25 kg/m^2^ as per validated population-based guidelines.^26^ Perioperative indices, including surgical approach and procedure, intraoperative blood loss, duration of hospital stay and number of harvested nodes, and postoperative outcomes were also recorded. The Clavien-Dindo stratification of surgical complications was used to classify morbidity with grade III or greater considered clinically significant.^27^ These were further subclassified into early (occurring within 30 days of surgery), intermediate (from 31 to 90 days), and late complications (beyond 90 days postoperatively).

Statistical analysis was performed using SPSS Statistics for Windows Version 23.0 (IBM Corp, Armonk, NY). Continuous variables (presented as mean and standard deviation) were compared using independent Student’s *t*-test (for two groups). To compare means of more than two groups, one-way ANOVA was used. Categorical variables were expressed as counts and percentages, chi-square test used to identify associations between these. When expected cell count was less than 5, Fisher’s exact test (two-sided) was performed. Binary logistic regression was used to determine the probability of an observation being associated with lymphocele development as a dichotomous dependent variable, first individually and following collinearity testing, as part of a multivariate model, selecting those predictors with *p*-value <0.1 on univariate analysis. For all other parameters, *p*-value <0.05 was considered statistically significant.

### Ethical statement

The study protocol adhered to the ethical standards of the Institutional Research Committee and with the Helsinki Declaration of 1975 and its subsequent amendments. Informed written consent was attained from all patients prior to any treatment or intervention. Since the study involved the retrospective analysis of anonymized data that had been obtained for clinical purposes, requirement for approval was waived by the local Ethics Committee. This study was conducted in accordance with the Strengthening the Reporting of Observational Studies in Epidemiology (STROBE) guidelines.

## Results

**Fig. 1:**
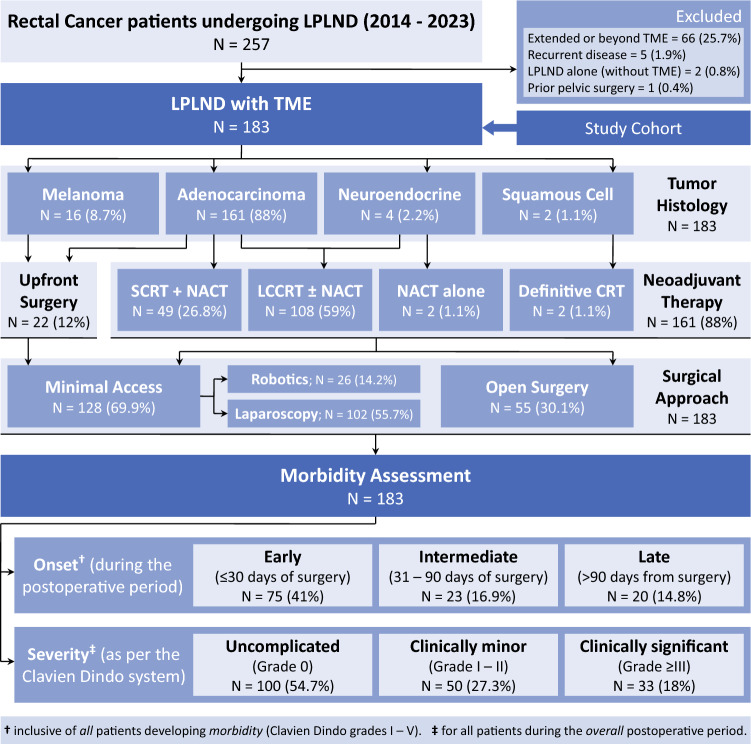
Study sample inclusion and exclusion criteria with the histological distribution, sequence of therapy and parameters considered for assessing postoperative morbidity in patients of the study population (n = 183). *CRT* chemoradiotherapy; *LCCRT* long-course chemoradiotherapy; *LPLND* lateral pelvic lymph node dissection; *NACT* neoadjuvant chemotherapy; *SCRT* short-course radiotherapy; *TME* total mesorectal excision

During the study period, 183 patients satisfying the inclusion criteria were selected for analysis (Fig. 1). Baseline clinicodemographic data and tumor characteristics have been summarized in Table 1.

**Table 1:** Baseline clinicodemographic features, treatment-related characteristics, and perioperative outcomes of patients in the study cohort (n = 183)

Characteristic			N	Value
Age (mean ± SD) in years			183	45.3 ± 12.81
Gender	Male		115	62.8%
	Female		68	37.2%
Performance status (based on ECOG)	Good (ECOG ≤ 1)		176	96.2%
	Marginal (ECOG ≥ 2)		7	3.8%
Tumor distance from anal verge (median, IQR) in cm			183	3.0 (1.0–5.0)
Body mass index (mean ± SD) in kg/m^2^			183	23.2 ± 4.02
BMI categories	Non obese (< 25 kg/m^2^)		132	72.1%
	Obese (≥ 25 kg/m^2^)		51	27.9%
Primary histology	Adenocarcinoma		161	88.0%
	Melanoma		16	8.7%
	Neuroendocrine carcinoma		4	2.2%
	Squamous cell carcinoma		2	1.1%
Neoadjuvant therapy	No		22	12.0%
	Yes	Radiotherapy ± chemotherapy	159	86.9%
		Chemotherapy alone	2	1.1%
Radiotherapy boost	No		128	80.5%
	Yes		31	19.5%
Surgical approach	Open		55	30.1%
	Minimal access	Laparoscopic	102	55.7%
		Robotic	26	14.2%
Surgical procedure	Low anterior resection		58	31.7%
	Intersphincteric resection		30	16.4%
	Abdominoperineal resection		95	51.9%
Laterality of LPLND	Unilateral		135	73.8%
	Bilateral		48	26.2%
Intraoperative Blood Loss (median, IQR) in ml			183	500 (250–800)
Re-exploration	Yes		12	6.6%
	No		171	93.4%
Hospital stay (median, IQR) in days			183	7.0 (5.0–9.0)
LPLN yield (median, IQR)	Unilateral		135	6.0 (4.00–9.00)
	Bilateral		48	11.5 (8.00–15.75)
Pathological LPLN status	Negative		132	72.1%
	Positive		51	27.9%
Postoperative morbidity	Clinically minor (CD grade I-II)		50	27.3%
	Clinically significant (CD grade ≥III)		33	18.0%
Pelvic lymphoceles (overall)	Clinically minor		26	14.2%
	Clinically significant		13	7.1%

*BMI* body mass index, *CD* Clavien-Dindo classification, *ECOG* Eastern Cooperative Oncology Group, *IQR* interquartile range, *LPLN* lateral pelvic lymph node, *LPLND* lateral pelvic lymph node dissection, *SD* standard deviation

Overall, 86.9% patients received neoadjuvant radiotherapy with or without chemotherapy of which 59% was LCCRT, dosed at 45–50.4 Gy in 25–28 fractions, 1.8–2 Gy/fraction with concurrent single-agent capecitabine, and SCRT in 26.8% (administered as 25 Gy in 5 fractions, 5 Gy/fraction with consolidation chemotherapy). The two patients with anorectal SCC had received 50 Gy in 25 fractions, 2 Gy/fraction, and additional boost of 10–15 Gy in 4–6 fractions (at 2.5 Gy/fraction) to the primary with concurrent capecitabine and mitomycin C prior to relapse. Of the four patients with neuroendocrine tumors, two received chemoradiotherapy and two chemotherapy alone. All patients with anorectal melanomas underwent upfront resection.

Surgery was performed between 8 and 12 weeks following radiotherapy and involved TME with unilateral or bilateral LPLND. The immediate postoperative period was uneventful in 59% of patients whose median duration of hospital stay was 6 (IQR 5–8) days. During the same period, 12.6% of patients were observed to have developed Clavien-Dindo grade III or greater morbidity, prolonging their hospital stay to median 12 (IQR 6–15) days. Overall, 12 patients required reexploration at a median of 6.5 days after primary surgery (IQR 4–16.75 days). Among the minor complications, acute urinary retention necessitating recatheterization was observed in 8.2%, of which five patients (2.7%) were on long-term clean intermittent catheterization at the end of the study.

**Fig. 2:**
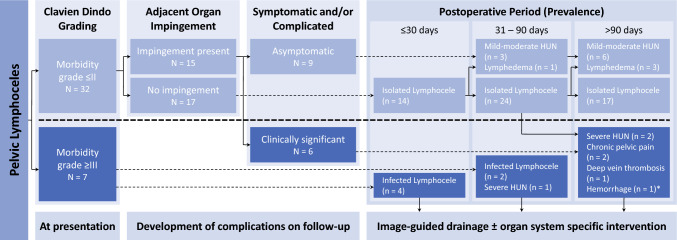
Associated morbidity and outcomes of lymphoceles encountered in patients of the study cohort at presentation and during follow-up in the early, intermediate and late postoperative periods

The overall incidence of lymphoceles during the median follow-up period of 13 (IQR 3–29) months was 21.3%, with 46.2% detected during the early postoperative period. The intermediate and late periods accounted for 43.6% and 10.3% of collections, respectively. Overall, seven patients (17.9%) were symptomatic at presentation because of secondary infection in six and gross hydroureteronephrosis in one; all of them were managed by image-guided drainage (with transurethral ureteric stenting in the latter). Among the 32 incidentally detected asymptomatic lymphoceles (comprising 82.1%), 38.5% were found to impinge on adjacent organs on follow-up, the severity of morbidity categorized as Clavien-Dindo grade III or greater in 15.4% (inclusive of one mortality). In the latter group developing delayed clinically significant collection-related complications, two patients with hydroureteronephrosis underwent cystoscopy-guided ureteral stenting and one required IVC filter placement for extensive thrombosis of the lower limb and pelvic venous systems. In two patients, lymphocele-associated chronic pelvic pain refractory to conservative measures was alleviated following percutaneous drainage. One patient experienced a Clavien-Dindo grade V complication; this 47-year-old man, who had received SCRT and chemotherapy for LARC prior to laparoscopic low anterior resection with unilateral LPLND, developed lymphocele-associated ipsilateral lower limb DVT in the intermediate postoperative period and was on enoxaparin when he presented with hemorrhagic peritonitis more than 3 months after surgery due to external iliac vein blowout, succumbing to hypovolemic shock and acute kidney injury despite reexploration and vascular control. The range of complications encountered, their grade, and the timeframe of occurrence in the postoperative period have been illustrated in Fig. 2 and summarized in Table 2.

**Table 2:** Spectrum of complications encountered with the corresponding Clavien Dindo grade, grouped by clinical-significance and occurrence in the early (within 30 days of surgery), intermediate (from 31 to 90 days), or late postoperative periods (beyond 90 days after surgery)

Postoperative period	Morbidity	Clavien-Dindo grading			
		Grade 0	Grade I–II	Grade III–V	Overall (Grade I–V)
Early (n = 183)	Overall	108 (59%)	52 (28.4%)	23 (12.6%)	75 (41%)
	Pelvic lymphoceles		14	4	18
	Surgical site infection		16	1	17
	Urinary complications^a^		16	–	16
	Bowel-related^b^		9	3	12
	Fascial dehiscence		1	7	8
	Stomal complications^c^		4	2	6
	Deep vein thrombosis		3	1	4
	Anastomotic leak		–	3	3
	Lymphedema		3	–	3
	Hemoperitoneum		–	2	2
Intermediate (n = 136)	Overall	113 (83.1%)	20 (14.7%)	3 (2.2%)	23 (16.9%)
	Pelvic lymphoceles		14	3	17
	Urinary complications^a^		7	1	8
	Lymphedema		1	–	1
Late (n = 135)	Overall	115 (85.2%)	12 (8.9%)	8 (5.9%)	20 (14.8%)
	Urinary complications^a^		6	4	10
	Pelvic lymphoceles		4	–	4
	Lymphedema		4	–	4
	Chronic pelvic pain		–	2	2
	Hemorrhage*		–	1	1
	Deep vein thrombosis		–	1	1

^a^Urinary complications included acute retention of urine, strictures, hydroureteronephrosis, urosepsis or renal failure

^b^Bowel-related complications included ileus, obstruction (regardless of etiology) or perforation

^c^Stomal complications included stomal retraction, prolapse or necrosis, and parastomal hernias

^*^Mortality

Binary logistic regression was performed to identify potential factors associated with the development of lymphoceles post-LPLND and on univariate analysis, obesity, neoadjuvant radiotherapy, open surgical approach, bilateral LPLND, nodal yield, and intraoperative blood loss were found to be statistically significant (Table 3). Estimation of variance inflation factors and tolerance, to measure the correlation and strength of association between these predictor variables, ensured that the assumption of multicollinearity was fulfilled. The multivariate regression model comprising obesity, receipt of radiation, open approach and nodal yield was statistically significant [χ^2^ (df = 6, n = 183) = 31.215, *p *< 0.001], explaining between 15.7% (Cox and Snell R^2^) and 24.3% (Nagelkerke R^2^) of the variance in the likelihood of patients developing these collections and correctly classifying 80.9% of cases (from 78.7%), with the Hosmer-Lemeshow test suggesting that the model was a good fit of the data [goodness-of-fit χ^2^ (df = 8, N = 183) = 8.022, *p *= 0.431] (Table 4).

**Table 3:** Univariate binary logistic regression analysis of potential factors predictive for the development of pelvic lymphoceles in patients undergoing LPLND for lateral pelvic node-positive rectal cancer (n = 183)

		Hazard ratio	95% Confidence intervals		*p*
			Lower	Upper	
Age		0.997	0.970	1.025	0.856
Sex		0.698	0.327	1.490	0.353
ECOG (status ≥2)		1.652	0.193	14.145	0.647
BMI (≥25 kg/m^2^)		2.166	1.031	4.555	**0.041**
Receipt of neoadjuvant RT		7.223	0.944	55.275	**0.057**
RT Protocol (LCCRT vs. SCRT)		0.992	0.453	2.173	0.984
Receipt of RT boost		1.389	0.523	3.688	0.510
Receipt of consolidation chemotherapy		1.195	0.520	2.746	0.675
Open surgical approach (vs. MIS)		3.278	1.573	6.832	**0.002**
Surgery	Low anterior resection				
	Intersphincteric resection	1.054	0.370	3.002	0.922
	Abdominoperineal resection	0.865	0.390	1.918	0.722
Bilateral LPLND		2.435	1.151	5.151	**0.020**
Total number of nodes harvested		1.078	1.019	1.140	**0.009**
Positive LPLN status		1.373	0.600	3.138	0.453
Intraoperative blood loss		1.000	1.000	1.001	**0.073**

Bold values are statistically significant at *p* < 0.1

*BMI* body mass index, *ECOG* Eastern Cooperative Oncology Group, *LCCRT* long-course chemoradiotherapy, *LPLN* lateral pelvic lymph node, *LPLND* lateral pelvic lymph node dissection, *MIS* minimally invasive surgery, *RT* radiotherapy, *SCRT* short-course radiotherapy

**Table 4:** Multivariate binary logistic regression of factors predictive for lymphocele development following LPLND for lateral pelvic node-positive rectal cancer, with the corresponding collinearity statistics (n = 183)

	Hazard ratio	95% Confidence interval		*p*	Collinearity statistics	
		Lower	Upper		Tolerance	VIF
BMI (≥ 25 kg/m^2^)	2.496	1.094	5.695	**0.030**	0.977	1.024
Receipt of neoadjuvant RT	10.026	1.225	82.027	**0.032**	0.924	1.082
Open surgical approach	2.779	1.202	6.425	**0.017**	0.853	1.172
Bilateral LPLND	1.266	0.487	3.293	0.629	0.761	1.314
Intraoperative blood loss	1.000	1.000	1.001	0.450	0.851	1.175
Total number of nodes harvested	1.105	1.026	1.190	**0.008**	0.746	1.341

Bold values are statistically significant at *p* < 0.05

*BMI* body mass index, *LPLND* lateral pelvic lymph node dissection, *RT* radiotherapy, *VIF* variance inflation factors

## Discussion

For locally advanced lateral node-positive malignancies of the rectum, chemoradiotherapy has been reported insufficient to address clinically significant pelvic lymphadenopathy, necessitating surgical clearance of the lateral pelvic nodal basins.^28,29^ Yet LPLND is a technically challenging procedure, more so in the post-radiation setting, with a significant learning curve and greater associated morbidity than TME alone.^9,24^ Most studies have reported outcomes associated with pelvic dissections for urological and gynecological malignancies.^1,30^ However, lymphadenectomies in these circumstances are commonly performed for cancer staging rather than radical nodal clearance and are often per primum with limited templates, hence differing in both indication for and extent of dissection.

In this study, the overall incidence of postoperative morbidity in patients undergoing TME with LPLND was 45.4% with 18% considered clinically significant and requiring intervention. In the latter group, 69.7% of complications (and 91.7% of reexplorations) were encountered within 30-days postprocedure and were predominantly on account of fascial dehiscence, intestinal obstruction, anastomotic leaks and stomal complications. This pattern of morbidity differed from the subsequent periods of follow-up when lymphocele-related mass effects, commonly obstructive uropathy and lymphovascular insufficiency, were more prevalent. Although clinically significant urinary complications were rare during the immediate postoperative period, the incidence of acute retention was comparable to data from other high-volume centers with experienced operators and may be attributed to the proportion of minimal-access procedures and unilateral LPLNDs performed, the exclusion of extended resections from the cohort, and the preservation of the inferior vesical pedicles and autonomic pelvic nerves when feasible during surgery.^31,32^ However, post-void residues or urodynamic studies to detect subclinical bladder dysfunction were not routinely performed.

Accounting for 47% of the overall morbidity and 39.4% of all clinically significant complications, lymphoceles comprised the single most common sequelae encountered following LPLND across the postoperative period. In accordance with literature, nearly 50% were detected within 30 days of surgery,^30,33^ partially explained by the higher incidence of symptomatic collections (22.2%) and coexistent complications (41%) during this period, many of which were radiologically evaluated. Since the majority of lymphoceles were incidentally detected and of Clavien-Dindo grades I and II, it is likely that the figures in this study underestimate the true incidence of these collections.

On univariate analysis of potential factors associated with lymphocele development, an increased predisposition was evident with open surgery, bilateral LPLND, greater nodal harvest, and higher blood loss, all surrogate indicators for operative complexity and more extensive dissection, the resulting lymphatic disruption, inflammation, and surgical dead space all contributing to a higher propensity to develop collections. Nonetheless, on the multivariate model, only open surgical approach and nodal yield retained predictive significance as the most potent of these factors, similar findings having been reported in gynecological and urological malignancies.^11–13,15,17,34^ At a center where minimal-access options were preferred for rectal cancer surgery, patients undergoing open resections may also have represented a cohort with potentially more heavily treated, advanced disease not amenable for a minimally invasive approach.

Although mean BMI was not significantly higher in patients with collections on account of outliers distorting the results, the dichotomized variable at a cutoff of 25 kg/m^2^ was found to be a significant predictor of lymphocele formation. Apart from the intraoperative challenges and delayed postoperative recovery associated with obesity, higher BMI has been reported to adversely affect lymphatic return, with the chronic low-grade perilymphatic inflammation leading to lymph stasis, increased capillary permeability and extravasation, predisposing to the development of lymphoceles.^11,35^

Radiotherapy has similar detrimental effects on lymphatic function. The inhibition of lymphatic proliferation and induction of nodal fibrosis results in impaired capillary patency and filtration capacity, giving rise to long-term often treatment-refractory sequelae.^36,37^ Greater tissue friability and interstitial edema in the early post-radiation period, evolves into fibrosis and fusion of tissue planes with increasing intervals to surgery, resulting in greater intraoperative blood loss, longer operative duration, and a higher propensity to develop lymphatic leaks. Node-positivity has been implicated as a strong predisposing factor for lymphocele formation in genitourinary malignancies.^11,16^ Yet, a similar association was not evident in this study, potentially owing to the field effect of radiotherapy on nodal tissue regardless of tumor involvement.

Although other studies have included histology-specific stage and pathological indices in their predictive models, the variables included in our regression analysis were restricted to clinicodemographic and surgery-associated intraoperative and postoperative parameters, since the primary objective of this study was to assess the surgical morbidity associated with LPLND. Also, because the steps of surgery and template for dissection remained uniform regardless of the pathological diagnosis, patients with histologies other than adenocarcinoma, which comprised 12% of the study population, were not excluded, resulting in the development of a more comprehensive model that could be applied independent of rectal cancer type. A histology-independent analysis also enabled us to evaluate the impact of neoadjuvant therapy specifically radiation in the development of lymphoceles.

In agreement with current literature, 7.1% of patients were observed to have developed clinically significant Clavien-Dindo grade III or greater morbidity on account of collections. The proportion of lymphoceles with complications was highest in the first month after surgery (22.2%).^15^ Of all patients with pelvic collections, 15.4% were observed to have developed morbidity in a metachronous manner following a variable asymptomatic interval. Lymphocele-related mass effects when causing adjacent organ impingement were most likely to involve the ureters. Conversely, the majority of urinary complications observed were associated with these collections, although cicatrization at the pelvic sidewall or ureteric devascularization secondary to LPLND could also have led to stricture-induced hydroureteronephrosis. Another source of significant morbidity following LPLND as evident in this study has been the dissection of major pelvic vessels in an irradiated field.

Periodic surveillance for lymphoceles in patients at risk post-LPLND has not been widely considered, possibly on account of its unfavorable cost-effectiveness, since the vast majority of collections remain undetected and regress spontaneously over time.^33^ In this study, of all asymptomatic lymphoceles, 12.5% progressed to develop clinically significant complications that required interventional radiological or surgical management, indicative of the potential yield derived from screening programs with the caveat that any prophylactic lymphocele-directed intervention would carry the risk of introducing secondary infection into an otherwise sterile collection and of reaccumulation.

### Limitations

Despite variations in radiotherapy dosing with histology and the treatment regimen followed, all radiation-treated patients were grouped together when performing logistic regression. As the strongest predisposing factor for lymphocele development in our analysis, further quantification of cumulative dose-adjusted risk was not performed. Although the surgeons participating in this study were considered to have negotiated the LPLND learning curve for either of the operative approaches, variations in lymphocele incidence over the course of the study period resulting from temporal increments in surgical experience as an operator-dependent variable was not evaluated further or included in the predictive model. Higher surgeon-volumes are known to improve perioperative patient-related outcomes that might have manifested as declining trends in operative duration, blood loss, and procedure-related morbidity (including lymphoceles) with time. An important predictor of collection-related complications, lymphocele volume was not estimated in this study, the irregular boundaries that such collections conform to precluding accurate measurement of their dimensions. Although proximity to vital anatomical structures may appear more clinically relevant than size alone, associated symptomatology and morbidity would likely remain a function of both these factors. As a significant limitation restricting the wider clinical applicability of the findings, risk factors predisposing to the development of complications in lymphoceles were not assessed on account of the small number of patients who developed clinically significant collections and the heterogeneity in patterns of morbidity encountered, both these features precluding a comprehensive and rational analysis. Lastly, asymptomatic lymphoceles were not considered for short-interval follow-up with dedicated imaging studies unless developing symptoms or signs suggestive of complications. In the absence of a formal protocol for lymphocele surveillance, the pattern of behavior in terms of progression or resolution over time could not be evaluated.

### Future Implications

Lymphoceles have been recognized as sequelae unique to LPLND. A range of intraoperative preventive strategies have been described in the literature to reduce their incidence, including the use of vessel-sealing energy devices and lymphatic clipping to minimize extravasation, the fenestration procedure and peritoneal flaps facilitating the drainage of contents into the peritoneal space, and measures to obliterate the dissection cavity by sclerotherapy, fibrin glue, or drain placement.^30^ Although the study model may facilitate the preemptive implementation of such interventional measures by prospectively identifying patients at increased risk of developing collections, perhaps of greater clinical relevance would be models capable of predicting the probability of complications arising in asymptomatic lymphoceles, the determinants of which would likely include collection volume, interval from surgery, progression on follow-up, and anatomical extent relative to adjacent organs. As a potential domain for future research, dedicated prospective studies with predetermined follow-up protocols would be instrumental to determine the margin of benefit offered by active lymphocele-directed surveillance and in formulating cost-effective screening strategies, thereby facilitating the individualization of patient-care.

## Conclusions

Clinically significant complications were observed in 18% of patients undergoing TME with LPLND, 39.4% of which were related to lymphoceles, the single most common sequelae encountered across the postoperative period. Obesity, neoadjuvant radiotherapy, open surgical approach, and nodal yield were significantly associated with the development of these collections.
